# Reappraisal of Pediatric Normal-Pressure Hydrocephalus

**DOI:** 10.3390/jcm10092026

**Published:** 2021-05-09

**Authors:** Owen P. Leary, Konstantina A. Svokos, Petra M. Klinge

**Affiliations:** 1 Department of Neurosurgery, The Warren Alpert Medical School of Brown University, Providence, RI 02903, USA; owen_leary@brown.edu (O.P.L.); konstantina.svokos@lifespan.org (K.A.S.); 2 Rhode Island Hospital, APC Building 6th Floor, 593 Eddy Street, Providence, RI 02903, USA

**Keywords:** pediatric hydrocephalus, normal-pressure hydrocephalus, neurodegenerative disease, congenital malformation, neurosurgery, pediatrics, ventriculoperitoneal shunting, cerebral palsy, cerebrospinal fluid, systematic review

## Abstract

While normal-pressure hydrocephalus (NPH) is most commonly diagnosed in older adulthood, a significant body of literature has accumulated over half a century documenting the clinical phenomenon of an NPH-like syndrome in pediatric patients. As in adult NPH, it is likely that pediatric NPH occurs due to a heterogeneous array of developmental, structural, and neurodegenerative pathologies, ultimately resulting in aberrant cerebrospinal fluid (CSF) flow and distribution within and around the brain. In this review, we aimed to systematically survey the existing clinical evidence supporting the existence of a pediatric form of NPH, dating back to the original recognition of NPH as a clinically significant subtype of communicating hydrocephalus. Leveraging emergent trends from the old and more recent published literature, we then present a modern characterization of pediatric NPH as a disorder firmly within the same disease spectrum as adult NPH, likely with overlapping etiology and pathophysiological mechanisms. Exemplary cases consistent with the diagnosis of pediatric NPH selected from the senior author’s neurosurgical practice are then presented alongside the systematic review to aid in discussion of the typical clinical and radiographic manifestations of pediatric NPH. Common co-morbidities and modern surgical treatment options are also described.

## 1. Introduction

The existence of a syndrome in children and young adults with presentation and clinical findings similar to normal-pressure hydrocephalus (NPH) has been acknowledged in the pediatric population [1,2,3,4,5,6,7,8,9,10,11,12,13,14,15] as a form of communicating hydrocephalus with normal intraventricular cerebrospinal fluid (CSF) pressure, resulting in progressive cerebral atrophy, ventricular dilation, and impairment of developmental cognitive and gross motor milestones [16,17]. In the adult or aging population, NPH is classified as “idiopathic NPH” or “secondary NPH,” with the latter associated with an antecedent event such as traumatic brain injury, stroke, hemorrhage, meningitis, or brain tumor [18,19,20,21]. The pathophysiology of both idiopathic and secondary NPH across the age spectrum is likely heterogeneous, based on age-related differences in cerebrospinal fluid physiology. While in the aging population, cerebrovascular incapacity and blood–brain barrier (BBB) disruption may play a role in the propagation of hydrocephalus, immature CSF absorption as well as incomplete skull formation are likely contributing factors in pediatric NPH.

Despite decades of recognition in the published literature, classification and diagnosis of NPH in the pediatric population remains controversial. The term “compensated ventriculomegaly” has been a misnomer applied in associated conditions which are linked to the development of chronic hydrocephalus which may or may not necessitate shunt treatment. Such conditions include prematurity, post-traumatic hydrocephalus, spina bifida, Chiari malformation, suboccipital pathologies (such as Dandy–Walker, retrocerebellar arachnoid cysts), benign extra-axial fluid collections in infancy, craniosynostosis, Down Syndrome or other syndromic conditions, storage diseases, meningitis, vascular malformation (e.g., Vein of Galen malformations) or other vascular complications [2,7,8,9,10,11,12,13,15,22]. Notably, all these bear a neurodegenerative component that links pediatric hydrocephalus to aging hydrocephalus pathologically and may be associated with insidious pattern of symptom onset following an initial insult. Further, age-related immaturity of CSF absorption and clearance similar could be considered analogous to age-related incompetence of CSF absorption in the older adult NPH population.

If one considers that the clinical hallmarks of NPH include the clinical triad (cognitive impairment, motor functional decline, and loss of bladder control) with symptomatic progression in the presence of chronic ventriculomegaly, or progressive cognitive and motor decline with or without urinary incontinence but in the absence of progressive ventriculomegaly, the literature collectively supports an NPH-equivalent diagnosis in the pediatric population. Most such situations where a similar clinical pattern is seen among children, often including failure to meet developmental milestones in lieu of marked deterioration of function per se, are associated with the above chronic conditions. Among both adults and children, presentation of cognitive dysfunction as well as treatment responsivity is widely variable, perhaps due to generally high prevalence of comorbidity with other brain pathology. Indeed, the possible importance of comorbid neurodegenerative pathology has recently emerged in the adult NPH literature [23,24,25,26]. As such, we postulate that there is likely a similar aspect which links pediatric and adult presentations, variably resulting from pre-existing injury, antecedent injury, or syndromic conditions. A commonality across these etiologies may also be a disruption of the CSF resorption at the BBB, which can occur with either prematurity or aging [2,5,27,28].

In this article, we aimed to systematically survey the literature evidence of pediatric NPH as a unique diagnostic entity which has emerged over the past five decades. Trends regarding etiology and diagnosis are qualitatively analyzed and discussed. Literature findings are coupled with a series of representative case presentations from the senior author’s clinical practice to support the concept of pediatric NPH applied in a modern cohort. Special consideration is paid to etiological circumstances or comorbidities of NPH in the pediatric population, and implications for clinical diagnosis and surgical management are highlighted.

## 2. Materials and Methods

**Figure 1:**
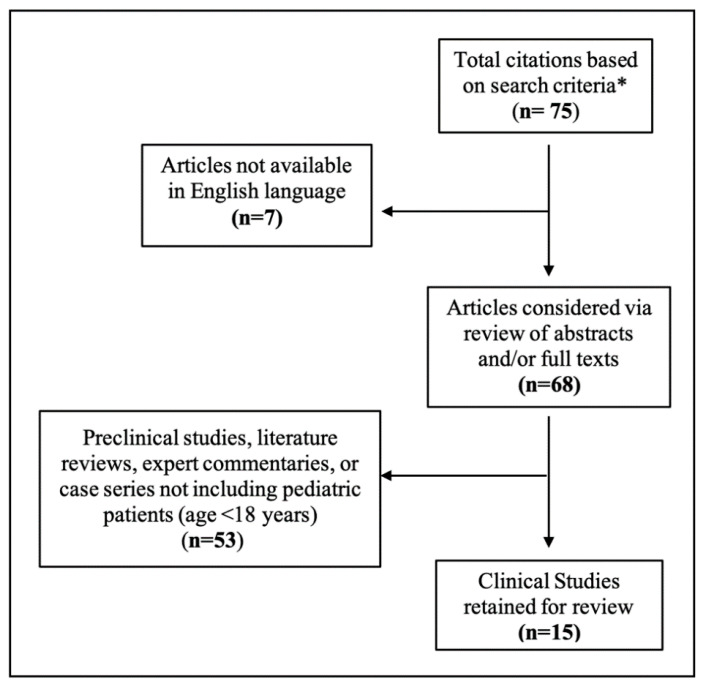
Results of systematic literature review. * Search criteria included ((Pediatric[Title/Abstract]) OR (Children[Title/Abstract]) OR (Child[Title/Abstract]) OR (Infant[Title/Abstract]) OR (Infants[Title/Abstract]) OR (Newborn[Title/Abstract]) OR (Newborns[Title/Abstract])) AND ((Normal Pressure Hydrocephalus[Title/Abstract]) OR (Idiopathic Hydrocephalus[Title/Abstract])).

We conducted a systematic search of the PubMed database (National Library of Medicine) to identify pertinent literature on the topic of pediatric NPH. Search terms included variants of “pediatric,” “child,” “infant,” or “newborn” in combination with “normal pressure hydrocephalus” or “idiopathic hydrocephalus,” contained within the title and/or abstract of the article. The citation lists of relevant articles identified by these criteria were additionally reviewed for studies which may have been excluded based on search criteria alone. This search methodology yielded a total of 75 PubMed-indexed articles for further examination. Abstracts and available full texts of identified articles were then individually reviewed for possible inclusion in this systematic review according to predefined criteria (Figure 1). Articles were excluded if they were not written or available in English language, or if they did not contribute new pediatric (age < 18 years) NPH cases to the literature. As such, examples of excluded studies are investigations and case series of NPH occurring only in adults (age > 18 for all subjects); studies of pediatric hydrocephalus not categorized or described by the authors as NPH or idiopathic hydrocephalus; literature reviews, meta-analyses, or expert commentaries; and cadaver or non-human animal studies. Level of evidence (I–V) was evaluated according to the Grades of Recommendation Assessment, Development, and Evaluation working group and the Agency for Healthcare Research and Quality (AHRQ) guidelines [29,30,31].

Our single-institutional case series spans eleven years of the senior author’s neurosurgical practice in pediatric hydrocephalus, spanning from 2009 to 2020. Electronic medical records were reviewed for cases consistent with the diagnosis of pediatric NPH, as described in the summarized literature. Nine cases were identified as illustrative examples of pediatric NPH, and key details including suspected or confirmed etiology, clinical presentation, radiographic findings, and treatment outcomes were tabulated. Six cases were selected for narrative case presentation vignettes (‘Case Presentations’).

## 3. Results

Review of the literature revealed 15 clinical studies describing unique cases of pediatric NPH, comprising a total of 146 patients ranging in age from infancy to teenage years. Included articles, along with key methodological details and results, are tabulated in Table 1. Overall, all articles offered only level III or IV evidence for the questions studied, comprising primarily single-institutional case reports, case series, and retrospective cohort studies. Primary research questions across these articles primarily related to the diagnosis, operative treatment outcomes (including ventricular shunting and/or endoscopic third ventriculostomy), and associated comorbidities of NPH in the pediatric population. Two articles included both pediatric and young adult patients with NPH or NPH-like syndromes. All articles found through this systematic review were published between 1972 and 2020.

Etiologically, pediatric NPH has been identified by the existing literature as emerging from a diverse range of degenerative, congenital, traumatic, and genetic conditions (Table 1). Common comorbid or historical factors frequently reported in association with these cases included prematurity, intraventricular hemorrhage, meningitis, prior radiation, brain trauma, and cerebral palsy [5,6,7,8,9,10,11]. Pediatric NPH has also been reported in connection with myelomeningocele and as a post-operative complication of posterior fossa surgery [14,15]. Some reports have also described idiopathic hydrocephalus in infants and children in which measured CSF pressure is within the expected range for the patient’s age [5,6,10]. Such studies have demonstrated that pediatric patients presenting with NPH from a variety of etiologies can be stratified in terms of CSF pressure, further corroborating the diagnosis of NPH in this population [6,10]. Consistent with prior hypotheses about disordered CSF production, recent reports and limited series have emerged highlighting specific genetic mutations which may be associated with the development of NPH early in life, in particular genes related to cilia motility [1,3]. Such results may have implications for the role of the choroid plexus in the pathophysiology of NPH in children, and potentially for adults. A larger body of work also exists, notably by Bateman and colleagues, demonstrating the possible role of altered venous flow dynamics in the development of NPH, as indicated by increased cerebral sinus stenosis and venous drainage through collateral flow pathways in patients with NPH and NPH-like presentations relative to controls [2,4,5].

The literature suggests that the presentation of children with NPH is similar to that of adults with NPH, frequently demonstrating at least two of the three of the classic triad of symptoms: progressive cognitive impairment, motor/gait disturbance, and urinary sphincter dysfunction. In children, progressive cognitive decline associated with NPH is frequently seen as failure to meet developmental milestones, sometimes such that overall progression lags behind verbal development if detailed neuropsychological testing is performed [6,10,14,15]. Similarly, gait disturbance may present as delay in motor milestones as well as spasticity. Gait, cognition, and urinary symptoms relative to developmental expectations may be challenging or impossible to evaluate in infants and newborns, such that reliance upon imaging evidence of communicating hydrocephalus and consistency of overall presentation with NPH may be necessary. Diagnostic criteria employed by each article are presented in Table 1.

### 3.1. Case Presentations

Nine illustrative cases from the senior author’s practice demonstrating the etiologic diversity and typical clinical presentation of NPH are tabulated in Table 2. Patient ages ranged from 12 months to 12 years at time of presentation. Five patients were male, and four were female. All underwent ventriculoperitoneal shunting (VPS) after neuroimaging findings of ventriculomegaly, with or without aqueductal narrowing, but otherwise with clearly non-obstructive hydrocephalus. The distribution of comorbid conditions and suspected etiologies across these cases was consistent with the published literature: 1 patient had Chiari malformation; 4 had underlying genetic defects with central nervous system effects (Pierre–Robin sequence; chromosomal duplication; Hunter syndrome); 2 were born prematurely (24 and 36 weeks); 1 had cerebral palsy secondary to cytomegalovirus; and 1 suffered an intrauterine stroke.

#### 3.1.1. Case #1. Hydrocephalus with Chiari I Malformation

**Figure 2:**
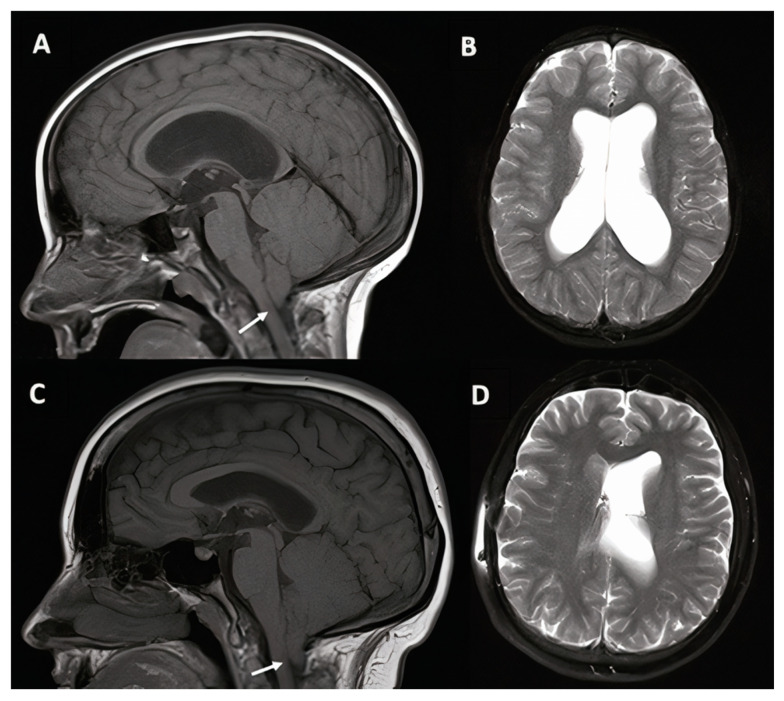
6-year-old female patient with Chiari I malformation (case #1). Pre-operative (**A**,**B**) and 10 years post-operative (**C**,**D**) T1-weighted midsagittal and T2-weighted axial magnetic resonance imaging (MRI). Inferior displacement of the cerebellar tonsils below the foramen magnum consistent with Chiari malformation is indicated by the white arrows.

A 6-year-old female patient with a known history of Chiari I malformation (CM1) with inferior displacement of the cerebellar tonsils presented with recent, progressive deterioration of school performance, increased distraction and irritability, and frequent falling, as well as recent worsening of occipital headaches attributed to CM1. The patient had also experienced recent occasional episodes of incontinence. MRI demonstrated ventriculomegaly (Figure 2). On exam, mental status change and impaired balance were also confirmed, demonstrating the full triad of NPH in a child. Neurocognitive evaluation supported diagnosis of attention deficit hyperactivity disorder (ADHD) and average performance in visual and verbal functions, suggestive of hydrocephalus and CM1 malformation. Given the cognitive implications and the gross motor decline, VPS was offered as the first treatment of choice. The procedure was uncomplicated and included the placement of a Codman–Hakim programmable valve. In the 10 years following surgery, the patient continued to experience intermittent headaches in the setting of asymmetrically drained ventricles without any sign of suboccipital headaches to suggest symptomatic Chiari malformation. Neurocognitive follow up over the years remained stable.

#### 3.1.2. Case #2. Congenital Hydrocephalus with Cerebral Palsy

**Figure 3:**
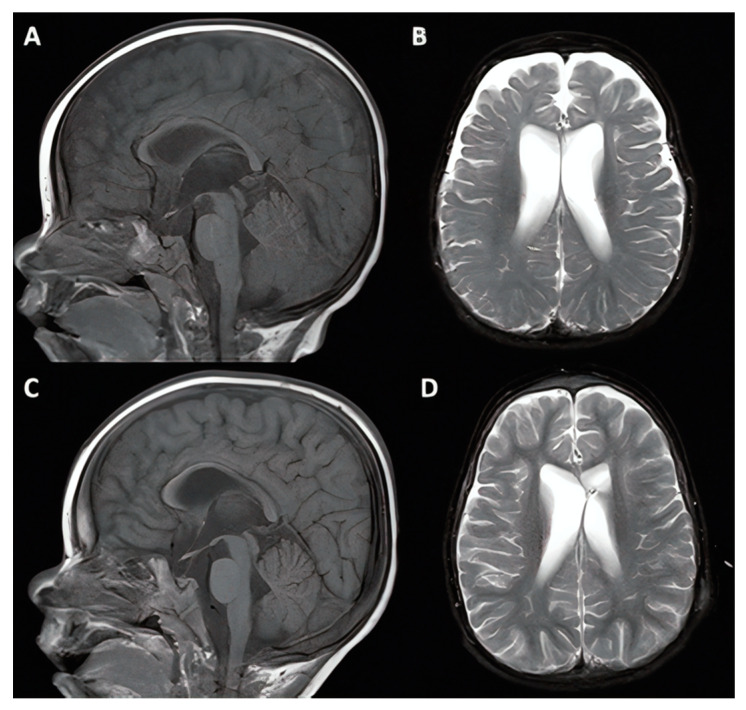
17-month-old male patient with cerebral palsy (case #2). Pre-operative (**A**,**B**) and 1 year post-operative (**C**,**D**) T1-weighted midsagittal and T2-weighted axial magnetic resonance imaging (MRI).

A 17-month-old male patient with cerebral palsy presented with a life-long history of developmental delay secondary to chronic lung disease and tracheobronchomalacia after premature birth at 24 weeks gestation. During a six-month stay in the neonatal ICU, the patient struggled with respiratory failure requiring months of respiratory support and was believed to have suffered anoxic brain injury early in life. The patient also had a history of episodic breath holding with cyanosis. The patient did not have any associated intraventricular hemorrhage based on serial CT evaluation. However, by 18 months, there was concern for incongruent motor and cognitive developmental delays, so VPS was offered in the setting of ventriculomegaly, and webbing of the cerebral aqueduct visualized on MRI. The patient had an uncomplicated procedure but experienced an inpatient course complicated by continued breath holding spells with episodes of bradycardia and was eventually discharged home after 1 week. The patient experienced continued psychomotor developmental delay and unfortunately was diagnosed with shunt infection and underwent VPS removal at 18 months following placement. The patient’s parents opted not to pursue further shunt placement given that the patient did not have any improvement. The patient required extensive support for ongoing cerebral palsy with associated spasticity and dystonia over the subsequent decade, but hydrocephalus remained stable with mild ventriculomegaly persistent through most recent MRI follow-up (Figure 3).

#### 3.1.3. Case #4. Congenital Hydrocephalus with Prematurity

**Figure 4:**
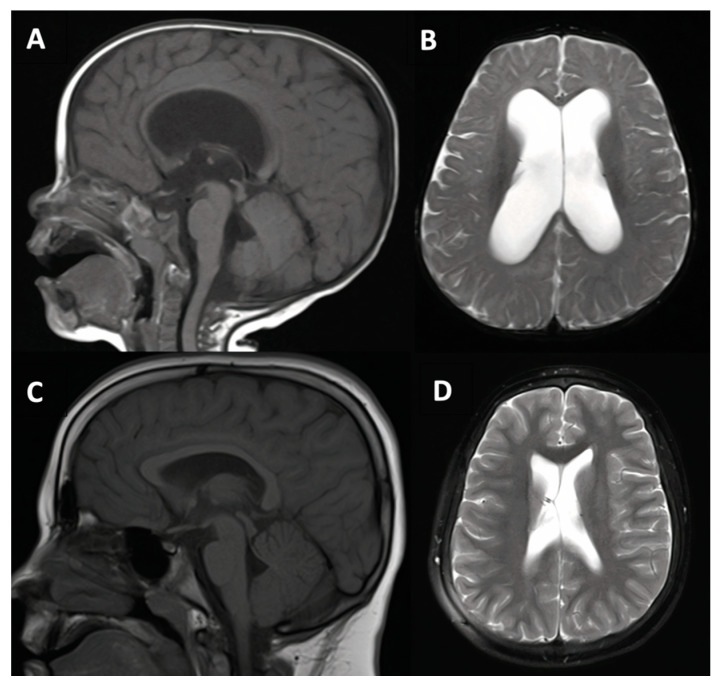
18-month-old female patient with premature birth and developmental delay (case #4). Pre-operative (**A**,**B**) and 8 years post-operative (**C**,**D**) T1-weighted mid-sagittal and T2-weighted axial magnetic resonance imaging (MRI).

An otherwise healthy 18-month-old female patient with history of premature birth (36 weeks) and being the smaller fraternal twin presented with delayed motor development relative to the twin first noticed as frequent tripping, falling, and unsteady gait. Mild spastic diplegia was noted on exam. Ultrasound and MRI studies both demonstrated ventricular enlargement. MRI additionally confirmed diagnosis of communicating hydrocephalus with possible partial aqueductal webbing (Figure 4). After discussion of treatment options, VPS was offered and completed without complication. A Codman–Hakim programmable valve was used. Post-operatively, the patient had a benign course for 9 years with gradual initial reduction in proportional ventricular size and then stable mild ventriculomegaly with resolution of symptoms except headache. An uncomplicated shunt revision was performed 9 years after the initial shunt in the setting of shunt malfunction.

#### 3.1.4. Cases #7–8. Hydrocephalus with Hunter Syndrome

**Figure 5:**
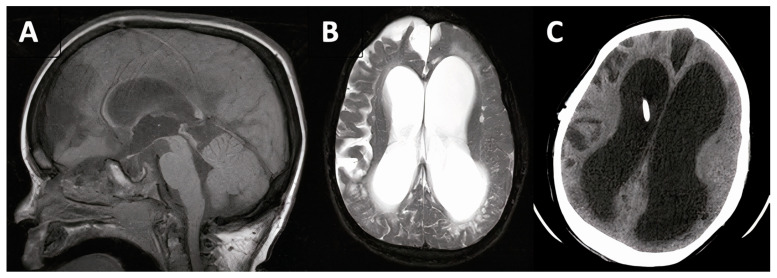
10-year-old male patient with Hunter syndrome and hydrocephalus (case #8). Pre-operative T1-weighted mid-sagittal and T2-weighted axial magnetic resonance imaging (MRI, **A**,**B**) and 1 year post-operative computed tomography scan with ventricular catheter visualized in the dilated right lateral ventricle (**C**).

Two male siblings with history of Hunter’s syndrome (including a third sibling who was diagnosed and passed away) presented at 10 and 12 years of age, respectively with progressive ventriculomegaly, cerebral atrophy consistent with neurodegeneration, and functional and motor deterioration. VPS was recommended in both cases. Both surgeries were completed uneventfully; in one, a Rickham reservoir was placed, and in the other, a Codman cylindrical valve. Both patients had post-operative difficulty with airway secretions and management but improved gradually and were each discharged within 3 days of surgery. The 12-year-old patient (case #7) was re-admitted two months later for headache, during which time he was diagnosed with an elbow abscess positive for staph aureus. Both patients experienced continued progressive decline as expected with Hunter’s syndrome over subsequent years but did not experience acute worsening of hydrocephalus or require shunt revision (Figure 5).

#### 3.1.5. Case #9. Congenital Hydrocephalus Following Intrauterine Stroke

**Figure 6:**
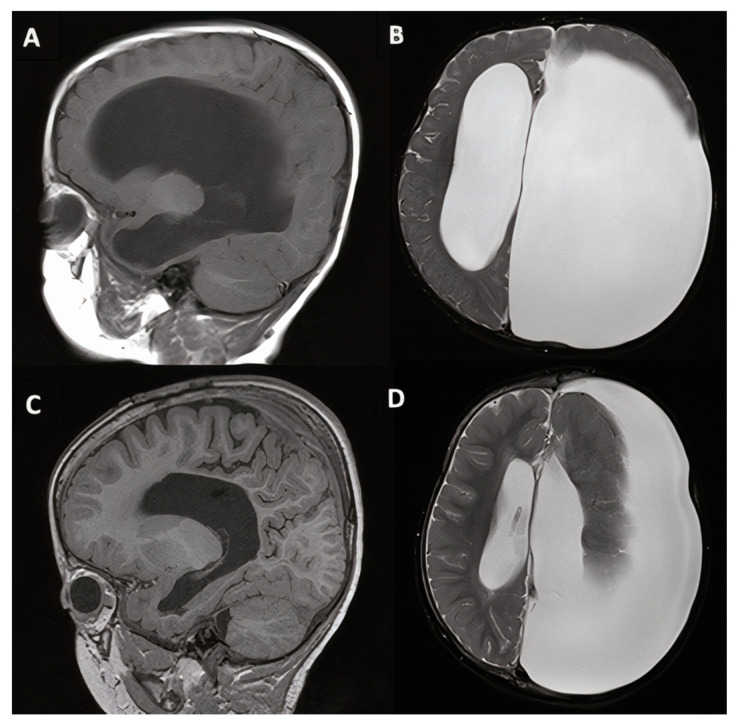
18-month-old male patient with left-sided porencephalic cyst secondary to intrauterine cerebral ischemia (case #9). Pre-operative (**A**,**B**) and 2 years post-operative (**C**,**D**) T1-weighted right-sided sagittal and T2-weighted axial magnetic resonance imaging (MRI), demonstrating ventriculomegaly as well as left-sided porencephalic cyst.

An 18-month-old male patient with history of intrauterine left-sided hemispheric stroke with residual cerebral ischemia and porencephalic cyst presented with interval history of slowing of motor development but continued verbal development. MRI demonstrated communicating hydrocephalus with massive left lateral ventricular enlargement with marked porencephaly, possibly dysplastic left-sided basal ganglia, and left-to-right midline shift (Figure 6). VPS was recommended, and the procedure took place with laparoscopic assistance for placement of the peritoneal catheter terminus. The patient did well neurologically but required three revision procedures over the first 6 months following initial VPS due to skin abscess progressing to eventual dehiscence and infection. His complicated post-operative course ultimately resulted in need for explanation of the original VPS, placement of an EVD, and subsequent placement of a right occipital VPS at 7 months after the original procedure. Certas PLUS valves were utilized. Subsequently, the patient went on to meet developmental milestones and begin pre-school without further need for revision or signs of shunt malfunction.

## 4. Discussion

Taken together, the results of the systematic literature review and institutional series comprise 155 total patients under the age of 18 years who underwent diagnosis and treatment of NPH-like syndromes. The findings of this systematic review help to establish the clinical phenomenology of a pediatric NPH-like syndrome. The 155 patients found through this review likely represent an under-estimation of the true number of pediatric cases published to date which are consistent with a diagnosis of NPH, as NPH diagnosis in this age group remains poorly defined and likely not often explicitly mentioned. Literature on this subject has also interestingly waned over the last two decades. These trends further underscore the need for additional study in support of evidence-based recommendations for this population. Based on published articles, the diagnosis and treatment of both secondary and idiopathic pediatric NPH is, in practice, comparable to that for adults, though without the same degree of standardization. That individual case series exhibit between-article variability in both etiology distribution and specific diagnostic criteria for NPH also highlights lack of standardization as far as when to suspect NPH in children. Establishment of pediatric NPH guidelines is warranted, similar to the guidelines that have been previously proposed for the adult NPH population [17,21].

### 4.1. Mechanisms of Pediatric NPH

While pediatric NPH is less likely to be mentioned explicitly in the more recent literature, some of the articles published in the last decade have provided novel mechanistic insights into potential genetic and circulatory pathophysiology present in children, as well as in adults, which may contribute to the development of NPH across ages. Two pediatric articles have implicated genes pertinent to ependymal ciliary structure and function; coupled with a third recent study by Yang et al. (2021) which observed a separate genetic defect also related to ependymal cilia function in 8/53 (15%) of adult idiopathic NPH patients who underwent full exosome DNA sequencing, this relatively new line of research provides insight into a potential mechanism of NPH related to cerebrospinal fluid production and circulation dynamics [1,3,32]. Separately, literature demonstrating altered cerebral blood flow dynamics in NPH supports one possible venous compensatory mechanism which may lead to ventricular dilation [2,4,5,28]. Finally, the hypothesis that NPH results from a CSF resorption problem at the level of subarachnoid granulations is also one with both old and new supporting evidence across age groups. The association between ventricular hemorrhage, subarachnoid inflammation, and the development of communicating NPH has been particularly well documented [7,9,10,11,22]. A more recent animal study of the transforming growth factor-β (TGF-β) antagonist decorin found that inhibiting TGF-β-induced subarachnoid fibrosis reduced ventricular enlargement in a rat model of juvenile communicating hydrocephalus [33]. Disordered glymphatic circulation and accumulation of macromolecules in the cerebral white matter has also been recently implicated in adult NPH [34,35]. These hypotheses about venous and cerebrospinal fluid circulation dynamics across the age spectrum are still emerging, but collectively support that NPH is likely not exclusively a condition of older age. These mechanisms likely all variably contribute to both “idiopathic” and secondary NPH in both children and adults, compounded by differences in brain and skull developmental status and resultant tissue compliance under pressure between age groups. As such, the etiology of NPH is probably highly heterogeneous, further underscored by such variable comorbid and historical associations seen across the patients studied.

Biomechanically, the impact of altered brain structure and neurodegeneration due to underlying comorbidities may also significantly predispose patients to developing NPH early in life, perhaps conceptually related to the high comorbidity of Alzheimer’s Disease-like pathology which has been appreciated in adult (but not pediatric) NPH [36,37,38,39,40]. While the overlap between childhood conditions resulting in cerebral malformation or neurodegeneration is not nearly as well studied as that between Alzheimer’s Disease and adult NPH, which may co-occur in up to 75% of some subsets of NPH patients [40], we did find anecdotal evidence of this phenomenon. Three articles included in our systematic review reported patients who had underlying congenital malformations, cerebral palsy or atrophy, premature birth, or syndromic condition besides hydrocephalus [8,10,13]. Two more described structural clinical correlates including myelomeningocele and posterior fossa surgery prior to NPH development [14,15]. Within our own case series, 9/9 (100%) of patients had some combination of these clinical features, including one with associated Chiari I malformation. While certainly requiring further investigation, the link between structural anomaly of the central nervous system and development of altered CSF dynamics resulting in non-obstructive hydrocephalus is not implausible. Furthermore, the other CSF circulatory anomalies mentioned in this review, including arachnoid granulation, choroid plexus, and glymphatic pathology, may perhaps overlap with structural distortion of the tissue in some of these complex patients.

As in adults, variation in CSF pressure dynamic profile across pediatric hydrocephalus etiologies has been documented previously [7,8]. Continuous ICP monitoring is a diagnostic tool of recognized importance in patients with chronic forms of hydrocephalus. Although baseline CSF pressure, and therefore individual pressure measurements, may be in the normal range in patients with chronic hydrocephalus, continuous monitoring may reveal more dynamic pressure alterations, such as “B waves” [41,42,43] as well as increased resistance to CSF outflow as documented in NPH (>13 mm Hg/mL per minute), which has helped to differentiate NPH from both brain atrophy with normal CSF circulation. Infusion studies in patients suffering predominantly from brain atrophy typically demonstrate low opening pressure, resistance to CSF outflow, and low pulse amplitude (ICP < 12 mm Hg, RCSF < 12 mm Hg/mL per minute, amplitude < 2 mm Hg) [8].

Continuous recordings have been previously applied to model and categorize communicating hydrocephalus in young adult patients, including those with congenital structural abnormalities underlying altered CSF dynamics such as myelomeningocele and leading to so called “arrested hydrocephalus” [44,45]. Given potential heterogeneity in pathophysiology and biomechanical underpinnings of NPH among pediatric patients across the age spectrum, continuous ICP recordings may be similarly useful in both the definitive diagnosis and understanding of pediatric NPH. It should be noted, however, that the practicalities of collecting continuous and reliable pressure recordings for such purposes are limiting in the pediatric setting.

### 4.2. Treatment and Outcome

Clinical and surgical decision making should be driven primarily by symptomatology, with clear evidence of deterioration or failure to meet milestones coupled with ventriculomegaly taken as a sign that surgical intervention may be indicated. In this series, all patients underwent VPS due to presentation including deterioration in some symptom domain consistent with NPH. Appropriately diagnosed and treated infants and young toddlers may be more likely to present chiefly with reduced global activity and progressive macrocephaly (e.g., cases #3, #4, #5, #9) while slightly older toddlers and children older than 4 may present with motor development outpacing verbal development, developmental slowing, or worsening of baseline neurologic symptomatology (e.g., cases #6, #7, #8). Older children are more likely to have more classic NPH-like symptomatology, such as in our 6-year-old female patient with Chiari I malformation who had deterioration of school grades (suggestive of cognitive slowing), balance, and urinary continence over a period of months (case #1). This patient was also diagnosed with ADHD and, leading up to VPS, reportedly became increasingly irritable. These symptoms were present at baseline and originally attributed to her Chiari I malformation but seemed to be exacerbated by progressing NPH. Somewhat similarly, case #7, a 12-year-old male with Hunter syndrome and baseline seizure activity, experienced an increase in seizure frequency prior to VPS. Such patients may benefit from close attention to progressive symptomatic worsening, and even mild early ventriculomegaly should be tracked closely with serial neuroimaging so as to discern more subtle but clinically significant presentations of NPH alongside existing neurologic comorbidities. Both of these patients experienced improvement after surgery, which consisted of ventriculoperitoneal shunt placement. It should also be noted that, as seen in adult hydrocephalus due to the neurodegenerative nature of NPH and frequently associated pathologies, patient symptoms can sometimes worsen despite VPS. This was unfortunately seen in two of our presented patients with baseline genetic comorbidities (cases #3 and #5), also with associated morbidity and mortality. While all nine pediatric NPH cases in this institutional series, and most cited from the literature, underwent VPS, endoscopic third ventriculostomy (ETV) is a viable evidence-based alternative in obstructive pediatric hydrocephalus, though evidence specifically in pediatric NPH is quite limited.

## 5. Conclusions

Normal-pressure hydrocephalus (NPH) remains a pertinent clinical diagnosis which is likely heterogeneous in pathophysiology and can occur in patients across the life cycle, including both older adults and children. The diagnosis of pediatric NPH is backed by less substantial clinical evidence than adult NPH. However, diagnosis and treatment across over 150 known patients, including those presented in this case series, has substantiated presenting clinical features similar to those in adult NPH including delay of cognitive, motor, and urinary developmental milestones during childhood. Ongoing study of genetic risk factors and neurodegenerative pathologies associated with NPH-like syndromes in children and adults may produce near-term mechanistic insight into this relatively common but likely underdiagnosed disorder. Ventriculoperitoneal shunting is a viable treatment option for patients with symptomatic pediatric NPH and the impact of endoscopic third ventriculostomy should be studied.

## Figures and Tables

**Table 1:** Summary of details from the published literature documenting pediatric normal-pressure hydrocephalus (NPH) or NPH-like presentation in children and young adults. References are numbered consistent with the citations list.

Reference	Study Design and Population	# of Patients	Key Findings, NPH Diagnostic Criteria, and Possible or Suggested Etiology	Evidence Level
Rocca et al., 2020 [1]	− Case report; genetic study − Infant (*n* = 1) with Primary Ciliary Dyskinesia (PCD)	1	Findings: A specific rare mutation was found in a patient with PCD who also presented with NPH, suggesting a new potential genetic risk factor Diagnosis: Ventriculomegaly on magnetic resonance imaging (MRI) without obstructive etiology Etiology: Mutation of Dynein Axonemal Intermediate Chain 2 (DNAI2), which is involved in cilia motility	4
Bateman et al., 2020 [2]	− Prospective comparative study (diagnostic) − Children with hydrocephalus (and age-matched controls) including a majority with communicating hydrocephalus (*n* = 36)	36	Findings: 19/36 patients with communicating hydrocephalus had significant venous sinus stenosis; venous sinus stenosis > 65% appears to be a marker of active (vs. compensated) hydrocephalus Diagnosis: 36 patients with communicating hydrocephalus as identified based on imaging studies indicating ventriculomegaly with at least some aqueductal flow Etiology: Heterogeneous etiologies (Table 2 in [2]) including infectious, congenital, and idiopathic cases	3
Kageyama et al., 2016 [3]	− Case series − Adults and infants (*n* = 5) with panventriculomegaly (PaVM)	5	Findings: PaVM is associated with an NPH-like presentation seen in both adults and children and DNAH14 mutations, and can be successfully treated with endoscopic third ventriculostomy when symptomatic Diagnosis: Ventriculomegaly with patent aqueduct, foramen of Magendie, and large cisterna magna as well as flow void Etiology: Mutation of dynein heavy chain protein gene DNAH14, which is functionally associated with cilia motility	4
Bateman, 2010 [4]	− Prospective comparative study − Children with idiopathic hydrocephalus and age-matched controls	9	Findings: Cerebral arterial flow was elevated, but sinus outflow was similar, in pediatric idiopathic hydrocephalus relative to age-matched controls; Increase in sinus pressure may lead to communicating hydrocephalus Diagnosis: Symptomatic idiopathic ventriculomegaly on imaging with (*n* = 3) or without (*n* = 6) aqueductal occlusion Etiology: Elevated cerebral sinus pressure leading to communicating hydrocephalus and possible increased, compensatory collateral venous outflow	3
Bateman et al., 2007 [5]	− Prospective comparative study − Children with idiopathic hydrocephalus (and age-matched controls)	14	Findings: Cerebral arterial flow was similar, but sinus outflow was reduced, in pediatric idiopathic hydrocephalus relative to age-matched controls. Diagnosis: Symptomatic idiopathic ventriculomegaly on imaging (*n* = 14) Etiology: Similar to adult patients with idiopathic intracranial hypertension, children with idiopathic hydrocephalus show significant elevation in collateral venous flow; ventricular dilation may depend on brain compliance in the presence of these altered flow dynamics	3
Crawford et al., 2000 [6]	− Case series − Children with hydrocephalus (*n* = 11), including some with idiopathic hydrocephalus (*n* = 3), and comorbid congenital heart disease	3	Findings: Of 11 children with hydrocephalus and congenital heart defects, 3 had idiopathic hydrocephalus. The population was characterized by developmental disabilities and high mortality rate (9/11 overall) Diagnosis: Symptomatic ventriculomegaly (without aqueductal stenosis or other obstructive cause, in idiopathic cases) Etiology: Not provided/evidenced; “idiopathic”	4
Bret et al., 1995 [7]	− Case series − Children with NPH	16	Findings: Among 16 patients < 20 years in age presenting with NPH, most displayed elements of the diagnostic triad, and intracranial pressure (ICP) was found to be within normal limits for 6/6 patients tested; shunting produced symptomatic improvement in 12/16 Diagnosis: ICP pressure measurement (6/6); adapted symptomatic triad, including psychomotor delay, gait abnormality, and urinary incontinence Etiology: Variable, including idiopathic, neonatal intraventricular hemorrhage, prior radiotherapy, aqueductal stenosis	4
Czosnyka et al., 1993 [8]	− Prospective comparative study (diagnostic) − Children with ‘arrested hydrocephalus’ (*n* = 115), including some classified by the study as having NPH (*n* = 18)	18	Findings: Among 115 cases of pediatric ‘arrested hydrocephalus’ described as hydrocephalus with non-progressing symptoms, 18 were classified as having NPH based on low resting cerebrospinal fluid (CSF) pressure and increased resistance to CSF outflow on lumbar infusion test; others could not be classified using these variables alone Diagnosis: Symptoms and computerized lumbar infusion test results Etiology: Factors frequently linked to the NPH group included birth trauma, head injury, infection, and congenital malformation (not further specified)	3
El Awad, 1992 [9]	− Retrospective case series and epidemiological study − Infants with hydrocephalus (*n* = 62), including some with congenital idiopathic hydrocephalus (*n* = 3)	3 (19)	Findings: Among 62 cases of infantile hydrocephalus identified in southwestern Saudi Arabia in a 3 year period, 3 had congenital idiopathic hydrocephalus, though 16 additional cases of “non-idiopathic” hydrocephalus were secondary to either cerebral hemorrhage or meningitis Diagnosis: retrospective epidemiological study (available hospital records) Etiology: Congenital idiopathic; 16 additional cases linked to pre- or postnatal hemorrhage or infection consistent with NPH-like etiology, but insufficient detail to be certain	4
Barnett et al., 1987 [10]	− Case series − Children and young adults with NPH	4	Findings: 4 cases in which NPH was identified in children and young adults underwent VPS with improvement in symptoms Diagnosis: Normal measured CSF pressure with ventriculomegaly, chronic or progressive neurologic deficits, and functional deterioration at presentation Etiology: Intraventricular hemorrhage, pre-mature birth w/cerebral palsy, meningitis, progressive cerebral atrophy	4
Torkelson et al., 1985 [11]	− Limited case series − Children with “arrested hydrocephalus” who benefitted from shunting, suggesting NPH	4	Findings: 4 cases in which pediatric hydrocephalus was not actively progressing and findings of sometimes subtle cognitive impairment on neuropsychology testing benefitted from shunting, suggesting sometimes subtle presentation of NPH in children Diagnosis: Ventriculomegaly with normal CSF pressure, neuropsychology testing consistent with cognitive impairment, and variable functional impairment and gait disturbances across the four patients Etiology: Variable, including idiopathic, meningitis, ventricular hemorrhage, and unknown due to unavailable early history	4
Hill and Volpe, 1981 [12]	− Case series − Premature infants with intraventricular hemorrhage (IVH, *n* = 87), including some with development of NPH (*n* = 20)	20	Findings: Of 87 patients born prematurely with intraventricular hemorrhage, 20 were found to develop NPH, highlighting these factors as potentially etiological in the development of NPH in children. Early recognition and suspicion can prevent harmful complications. Some cases may progress while others do not Diagnosis: Ventriculomegaly with serial imaging and correlation with symptoms Etiology: Prematurity with IVH	4
Brumback et al., 1978 [13]	− Limited case series − Pediatric patients with Cockayne’s syndrome and NPH	4	Findings: 4 cases of children presenting with Cockayne’s syndrome also present with symptomatic NPH, suggesting possible comorbidity between these disorders Diagnosis: Ventriculomegaly with developmental delay during childhood and gait disturbance, urinary incontinence, and/or psychomotor delay; normal CSF pressure in patients who were tested (2/4) Etiology: Premature birth with progressive neurologic anomalies classic to Cockayne’s syndrome	4
Hammock et al., 1976 [14]	− Case series − Children with myelomeningocele presenting with NPH	8	Findings: NPH-like presentation was identified in 8 patients with ventricular enlargement and known myelomeningocele, suggesting possible comorbidity related to flow dynamics; improvement after shunting Diagnosis: Ventriculomegaly with failure to keep up with developmental milestones on detailed neuropsychological testing (verbal outpacing overall performance), and improvement after shunting Etiology: Possibly related to flow dynamics in the setting of underlying myelomeningocele	4
Stein et al., 1972 [15]	− Limited case series − Children with NPH after undergoing posterior fossa neurosurgical procedures	3	Findings: NPH as a subtle complication of posterior fossa surgery is a recognizable post-operative event which can be successfully treated with shunting Diagnosis: Symptomatic, progressive post-operative ventriculomegaly with triad of symptoms similar to adult; normal CSF pressure Etiology: Possibly related to altered flow dynamics secondary to posterior fossa surgery	4

**Table 2:** Summary of details from nine cases consistent with pediatric NPH from the authors’ institution.

Case #	Age and Sex	History and NPH-Like Presentation	MRI Findings	Outcome and Clinical Course Following VPS Placement
1	6 y, F	− Chiari I malformation, ADHD − Irritability, behavior changes, deterioration of school grades, balance, urinary continence	− Ventriculomegaly − 7 mm inferior displacement of cerebellar tonsils	− Stable ventricular size without shunt malfunction at 10 years post − Ongoing, fluctuating symptoms of CM1 with improvement in ADLs
2	17 m, M	− Premature birth (24 w) w/cerebral palsy, hypoxia, bronchomalacia − Developmental delay	− Ventriculomegaly − Aqueductal webbing	− Interval improvement on MRI with VPS removal at 18 months post-op − Stable mild ventriculomegaly with psychomotor developmental delay
3	20 m, M	− Pierre–Robin sequence w/central apnea − Visual impairment, papilledema, developmental delays	− Ventriculomegaly − Extra-axial fluid accumulation − Aqueductal narrowing	− Stable ventriculomegaly with some symptomatic improvement − Death at 16 months following sepsis-induced cardiac arrest
4	18 m, F	− Fraternal twin with premature birth (36 w) − Motor developmental delay; mild macrocephaly	− Ventriculomegaly − Aqueductal webbing	− Gradual reduction in ventricular size on serial post-op imaging − Uncomplicated shunt revision 9 years post-op w/stable ventricles
5	12 m, F	− Chromosomal duplication syndrome; central apnea − Progressive macrocephaly	− Significant Ventriculomegaly − Progressive cerebral cortical thinning across serial scans	− Successful VPS − Death at 2 months post-op after enterovirus infection, systemic organ failure, and cardiac arrest
6	3 y, F	− Congenital cytomegalovirus, cerebral palsy, blindness, − Seizures increasing in frequency, severe motor and verbal developmental delay	− Ventriculomegaly − Cerebellar vermis hypoplasia; Dandy–Walker variant; corpus callosum and cortical malformation − Aqueductal narrowing	− Successful VPS without complication, stable compensated ventriculomegaly on post-op MRI − Lost to follow-up at 5 years post-op
7	12 y, M	− Hunter syndrome, sleep apnea − Increased head turning and crying; seizures increasing in frequency; functional decline	− Progressive Ventriculomegaly − Cerebral cortical thinning	− Difficult post-op course including respiratory difficulty and elbow abscess but without VPS infection − Stable ventriculomegaly
8	10 y, M	− Hunter syndrome − Recent functional and motor decline	− Progressive Ventriculomegaly − Cerebral cortical thinning	− Successful VPS with difficult post-operative course, including respiratory difficulty − Stable ventriculomegaly
9	18 m, M	− Hemispheric intrauterine stroke − Progressive macrocephaly, long-standing motor delay, global developmental slowing	− Ventriculomegaly − Left-sided porencephalic cyst secondary to cerebral ischemia	− VPS with complex post-operative course including infection, dehiscence, and shunt revision − Improved developmental progress
